# Biofilm Formation in Chronic Infections: A Comprehensive Review of Pathogenesis, Clinical Implications, and Novel Therapeutic Approaches

**DOI:** 10.7759/cureus.70629

**Published:** 2024-10-01

**Authors:** Kaushik Sahoo, Supriya Meshram

**Affiliations:** 1 Department of Microbiology, Jawaharlal Nehru Medical College, Datta Meghe Institute of Higher Education and Research, Wardha, IND

**Keywords:** antimicrobial resistance, biofilm-associated infections, biofilms, chronic infections, novel therapeutic approaches, phage therapy

## Abstract

Biofilms are intricate microbial communities on various surfaces, including medical devices and biological tissues, encased within a protective matrix of extracellular polymeric substances. Their formation and persistence are significant factors in the pathogenesis of chronic infections, contributing to the complexity of treatment and increased resistance to antimicrobial agents. This review explores the multifaceted nature of biofilms, focusing on their formation, structure, and the genetic and environmental factors that contribute to their resilience. Biofilms are particularly problematic in chronic infections, such as those associated with medical implants and persistent wounds, due to their ability to evade both the host immune response and conventional therapeutic strategies. The review also discusses the current challenges in diagnosing biofilm-associated infections and the limitations of existing treatment options. Emerging therapeutic approaches, including novel antibiofilm agents, physical disruption techniques, and biological therapies such as phage therapy, are examined for their potential to improve treatment outcomes. Innovations in drug delivery systems and preventive measures, such as biofilm-resistant materials, are also highlighted as promising developments. This comprehensive overview aims to provide insights into the mechanisms of biofilm-related infections and to guide future research and clinical practice. This review contributes to the ongoing efforts to enhance patient care and combat the growing challenge of antimicrobial resistance by addressing the critical need for effective strategies to manage and prevent biofilm-associated chronic infections.

## Introduction and background

Biofilms are complex communities of microorganisms that adhere to surfaces and are encased within a self-produced matrix of extracellular polymeric substances (EPS) [1]. This matrix, composed of proteins, polysaccharides, and nucleic acids, forms a protective barrier around the microbial cells, enabling them to thrive in hostile environments. Biofilms can form on biotic surfaces, such as tissues and organs, and abiotic surfaces, including medical devices and industrial equipment [2]. Forming biofilms involves well-defined stages: initial attachment, microcolony formation, maturation, and dispersion. During these stages, microorganisms communicate through quorum sensing, coordinating their behavior and enhancing the biofilm's resilience [3].

Biofilms are of significant concern in chronic infections due to their role in persistence and resistance. In chronic infections, biofilms can establish themselves in various body sites, including wounds, bones, and implants [4]. The biofilm matrix protects the microorganisms from host immune responses and antimicrobial agents, leading to prolonged infection and complicating treatment efforts. For example, biofilms on medical devices such as catheters or prosthetic joints can result in severe and persistent infections that are difficult to eradicate. The ability of biofilms to harbor multidrug-resistant bacteria further exacerbates the challenge, making these infections a major concern for both patient outcomes and public health.

This review aims to provide a comprehensive understanding of biofilm formation in chronic infections, focusing on three main areas: the pathogenesis of biofilm formation, the clinical implications of biofilms in chronic infections, and novel therapeutic approaches for their management. By exploring the mechanisms underlying biofilm development and persistence, this review highlights the challenges associated with biofilm-related infections and the limitations of current treatment strategies. Additionally, it will examine emerging therapeutic options and innovative approaches designed to disrupt or eradicate biofilms. Ultimately, this review aims to inform and guide future research and clinical practice in managing biofilm-associated chronic infections, offering insights into effective strategies for prevention and treatment.

## Review

Methodology

A comprehensive literature search was conducted in PubMed, Scopus, Web of Science, and Google Scholar using keywords such as “biofilm formation,” “chronic infections,” “pathogenesis,” “clinical implications,” and “novel therapeutic approaches,” focusing on articles published in English from 2000 to 2024. Eligible studies included original research, systematic reviews, and meta-analyses related to biofilm-associated chronic infections and therapeutic interventions. In contrast, non-English articles, conference abstracts, and low-quality studies were excluded. Data from selected studies were extracted and synthesized narratively, focusing on biofilm mechanisms, clinical implications, and therapeutic strategies. Quality assessment was conducted using appropriate tools, and findings were categorized into thematic areas, with descriptive analysis highlighting key trends. No ethical approval was required as the review was based on previously published studies, and potential limitations include study heterogeneity and language restrictions.

Pathogenesis of biofilm formation

Biofilm formation is a multifaceted process influenced by various microbial dynamics, including the types of microorganisms involved, the stages of development, and the genetic and environmental factors that govern this phenomenon [5]. Various microorganisms, including bacteria, fungi, and even viruses, can produce biofilms. Among bacteria, *Pseudomonas aeruginosa* and *Staphylococcus aureus* are two of the most frequently implicated species. *P. aeruginosa*, a Gram-negative bacterium, is particularly notorious for its ability to form resilient biofilms on various surfaces, including medical devices and the lungs of patients with cystic fibrosis [5]. This ability to form biofilms contributes to chronic infections and resistance to treatment. Likewise, *S. aureus*, a Gram-positive bacterium, is commonly associated with biofilms on indwelling medical devices and chronic wounds. Other species, such as *Escherichia coli*, also play a major role in biofilm formation, particularly in urinary tract infections [6]. In addition to bacterial biofilms, fungal biofilms, most notably those formed by Candida species, are clinically significant. These fungi can develop biofilms on medical devices such as central venous catheters and prosthetic heart valves, complicating treatment efforts [6]. The biofilm formation process is divided into several distinct stages. It begins with initial attachment, where planktonic (free-floating) microorganisms reversibly adhere to a surface via weak van der Waals forces and hydrophobic interactions. This phase is crucial for the subsequent stages [5]. Next comes irreversible attachment, in which the microorganisms produce EPS that enable them to form stronger, more permanent connections to the surface and one another. This leads to the maturation stage, during which the biofilm develops into a complex, three-dimensional structure with distinct microenvironments. The EPS matrix provides structural integrity and shields the microorganisms within from environmental stressors and antimicrobial agents. The final stage is dispersal, during which cells detach from the biofilm, either as individual cells or in aggregates, returning to a planktonic state and enabling the biofilm to colonize new surfaces [7]. Both genetic and environmental factors play key roles in regulating biofilm formation. Genetically, quorum-sensing is a crucial mechanism that allows bacteria to communicate and coordinate their behavior based on population density, influencing the expression of genes related to biofilm formation. Specific biofilm-associated genes are responsible for EPS production, attachment, and the overall architecture of the biofilm [8]. Environmental factors are equally important. Surface properties such as hydrophobicity and roughness can affect the initial attachment of microorganisms. Nutrient availability is critical for biofilm maturation, as microorganisms need sufficient resources to thrive. Factors such as temperature and pH influence biofilm development, with optimal conditions necessary for effective formation. Finally, hydrodynamic conditions, such as liquid flow over the surface, can impact the rate of biofilm formation and its structural characteristics [9]. Table 1 provides an overview of the stages and factors involved in biofilm pathogenesis.

**Table 1:** Stages and factors in the pathogenesis of biofilm formation EPS: extracellular polymeric substances

Stage	Description	Key processes involved	Examples of involved microbes
Initial attachment [10]	Bacteria or fungi attach to a surface (biotic or abiotic). The attachment is reversible initially but becomes irreversible over time	Surface adhesion molecules, pili, flagella, and hydrophobic interactions	*P. aeruginosa* and *S. aureus*
Microcolony formation [11]	After attachment, the cells start to multiply, forming clusters or microcolonies. The production of EPS begins	Quorum-sensing and EPS secretion	*E. coli* and *Candida albicans*
Maturation [12]	Biofilm matures into a complex 3D structure with distinct layers and nutrient gradients. Cells in the deeper layers become metabolically dormant	Nutrient gradient formation, biofilm architecture development, and metabolic heterogeneity	*P. aeruginosa* and *Staphylococcus epidermidis*
Dispersal [13]	Cells are released from the biofilm, actively or passively, to colonize new environments. This can occur due to environmental stress or nutrient depletion	Enzymatic degradation of EPS, detachment, and cell motility	*S. aureus* and *P. aeruginosa*
Genetic factors [14]	Genetic mechanisms controlling biofilm formation, such as quorum-sensing and biofilm-associated genes	Quorum-sensing, mutations in regulatory genes (e.g., LasR in Pseudomonas), and horizontal gene transfer	*P. aeruginosa* and *S. aureus*
Environmental factors [15]	Factors in the external environment affect biofilm formation, such as surface properties, nutrient availability, and temperature	Hydrophobicity, pH, nutrient concentrations, temperature, and oxygen availability	*P. aeruginosa* and *Streptococcus mutans*

Clinical implications of biofilms

Biofilms play a significant role in chronic infections, presenting numerous clinical challenges due to their ability to resist treatment and evade the immune system. Their involvement in persistent infections, particularly chronic wound infections and osteomyelitis, is especially concerning. In chronic wound infections, biofilms often form in nonhealing wounds, resulting in prolonged inflammation and resistance to standard treatments [16]. This complicates the healing process and frequently necessitates advanced therapeutic interventions, such as surgical debridement. Similarly, osteomyelitis, commonly associated with biofilm formation, can persist in bone tissue, especially in cases involving implanted devices or postsurgical infections. Biofilms in these situations lead to chronic osteomyelitis, which is notoriously difficult to eradicate [17]. Biofilm-associated infections linked to medical devices further underscore their clinical significance. Approximately 65% of device-related infections are attributed to biofilm formation [6]. For example, biofilms can develop on central venous catheters and prosthetic joints, causing serious complications such as bloodstream infections and periprosthetic joint infections. Treating such infections often requires removing the infected device, emphasizing the importance of preventive strategies in clinical settings [18]. One of the most serious complications of biofilm-associated infections is antimicrobial resistance. Bacteria within biofilms exhibit significantly greater antibiotic resistance than their planktonic counterparts, making treatment extremely challenging. This resistance arises from several factors, including altered bacterial metabolism, where reduced metabolic activity in biofilms leads to decreased antibiotic susceptibility. Additionally, persister cells, dormant variants within the biofilm, can survive antibiotic treatments and reactivate once therapy is discontinued, contributing to infection recurrence [19]. Biofilms also complicate immune system responses through various evasion mechanisms. The EPS matrix surrounding biofilms is a barrier, protecting the bacteria from immune cells and antimicrobial agents. Moreover, biofilms can trigger a chronic inflammatory response, further hindering the host’s ability to eliminate the infection and causing tissue damage and persistent symptoms [20]. Diagnosing biofilm-associated infections presents unique challenges as well. The symptoms often overlap with those of other conditions, making diagnosis difficult. Traditional microbiological methods may fail to detect biofilm-associated organisms, as these tests typically rely on the growth of planktonic bacteria rather than biofilm communities [21]. Advanced imaging modalities, such as ultrasound and MRI, can help visualize biofilm-related complications, especially in cases involving implanted devices. Additionally, tissue biopsy followed by molecular techniques, such as polymerase chain reaction, can accurately identify biofilm-forming pathogens, enabling targeted treatment strategies [21]. Table 2 outlines the clinical implications of biofilm formation in chronic infections.

**Table 2:** Clinical implications of biofilm formation in chronic infections UTIs: urinary tract infections; VAP: ventilator-associated pneumonia

Clinical aspect	Description	Biofilm-associated infections	Impact on treatment
Chronic infections [5]	Biofilms are a major cause of persistent infections, providing a protective environment for pathogens to evade immune responses and resist treatment	Chronic wound infections, osteomyelitis, chronic sinusitis, and cystic fibrosis lung infections	Difficult to eradicate, requiring prolonged antibiotic therapy and potentially surgical intervention
Medical device-associated infections [22]	Biofilms commonly form on implanted medical devices, leading to device-related infections often resistant to antibiotics and host defenses	Catheter-associated infections, prosthetic joint infections, and heart valve infections (endocarditis)	Often necessitates device removal or replacement, alongside targeted antimicrobial therapy
Antimicrobial resistance [23]	Biofilms enhance the resistance of microbes to antibiotics by reducing drug penetration and promoting the survival of resistant strains	Multidrug-resistant *P. aeruginosa* and *S. aureus* in biofilms on medical devices	Requires combination therapies and higher doses of antibiotics; increased risk of treatment failure
Immune system evasion [5]	The biofilm matrix protects pathogens from immune cells, making it difficult for the immune system to clear the infection	Chronic otitis media and UTIs caused by biofilm-forming bacteria on catheters	Leads to recurrent or persistent infections despite immune activation; promotes chronic inflammation
Diagnostic challenges [24]	Biofilm infections are often difficult to diagnose due to their resistance to culture techniques and nonspecific symptoms	Prosthetic joint infections, infections in cystic fibrosis, and endocarditis with biofilm-producing bacteria	Requires advanced diagnostic methods such as molecular techniques, imaging, or biopsy for accurate detection
Complications and outcomes [25]	Biofilm-associated infections can lead to severe complications, prolonged hospital stays, and increased mortality rates in vulnerable populations	Infected venous catheters, chronic osteomyelitis, and VAP	Poor clinical outcomes, increased healthcare costs, and higher risk of complications like sepsis or systemic spread

Novel therapeutic approaches

Novel therapeutic strategies to combat biofilm-associated chronic infections span a variety of approaches, including pharmacological interventions, physical and mechanical disruption, biological therapies, innovative drug delivery systems, and preventive measures. These strategies are designed to address the unique challenges posed by biofilms, which are notoriously resistant to conventional treatments [26]. Pharmacological interventions include the use of antibiofilm agents and combination therapies. Enzyme-based treatments, such as DNases, target the extracellular DNA within the biofilm matrix, disrupting its structural integrity and enhancing the efficacy of antibiotics. Additionally, various antibiofilm compounds, such as antimicrobial peptides like nisin, have shown promise in breaking down biofilms and increasing the susceptibility of biofilm-associated bacteria to traditional antibiotics [27]. Combination therapies pair antibiotics with biofilm-disrupting agents and have demonstrated significant potential in improving treatment outcomes by increasing drug penetration and effectiveness against bacteria embedded in biofilms [27]. Physical and mechanical disruption techniques are also critical for managing biofilm-related infections. One innovative method involves using ultrasound, which disrupts biofilm structures through cavitation. This process generates shock waves that physically break apart the biofilm, making the bacteria more vulnerable to antimicrobial agents. Surgical removal of biofilms may be necessary in severe cases, especially in device-associated infections. Surgical interventions to remove biofilm-contaminated devices eliminate the source of infection, allowing antibiotics to work more effectively [28].

Biological therapies, particularly phage therapy, offer another promising approach to combating biofilm-associated infections. Bacteriophages, viruses specifically targeting and lyse bacteria, can selectively attack biofilm-forming pathogens without harming beneficial microbiota. Phage therapy becomes even more effective when combined with enzymes that degrade biofilm components. This dual approach targets both the bacterial cells and the protective biofilm matrix, increasing the likelihood of successfully eradicating the infection [29]. Innovative drug delivery systems are essential for improving the effectiveness of biofilm treatments. Nanoparticle-based systems can be engineered to deliver antimicrobial agents directly to biofilm sites, ensuring higher local concentrations while minimizing systemic exposure [30]. Localized delivery methods, such as implantable devices that release antibiotics or biofilm-disrupting agents at the injection site, can provide sustained and targeted treatment. These advanced delivery systems enhance therapeutic outcomes by ensuring antimicrobial agents effectively reach the bacteria [30]. Preventive measures are equally crucial in the fight against biofilm-associated infections. Developing biofilm-resistant materials is key to preventing infections linked to medical devices. Antiadhesive surface coatings significantly reduce the risk of biofilm formation [31]. Additionally, surface modification technologies that alter implants' and medical devices' physical and chemical properties further decrease bacterial adhesion. By focusing on prevention, healthcare providers can reduce the incidence of biofilm-related infections and improve patient outcomes [31]. Table 3 highlights novel therapeutic approaches for biofilm-associated infections.

**Table 3:** Novel therapeutic approaches for biofilm-associated infections UTIs: urinary tract infections

Therapeutic approach	Description	Therapies	Clinical applications
Pharmacological interventions [7]	Drugs and compounds specifically targeting biofilms can be used by disrupting biofilm structure or enhancing the activity of conventional therapies	Enzyme-based therapies (e.g., DNase to break down extracellular DNA) and antibiofilm compounds (e.g., dispersin B)	Chronic wound infections, catheter-associated infections, and persistent infections such as osteomyelitis
Combination therapies [32]	Use of antibiotics in combination with biofilm-disrupting agents to enhance efficacy and reduce antimicrobial resistance	Antibiotics combined with biofilm-dispersing agents like lactoferrin or quorum-sensing inhibitors	Treatment of biofilm-associated infections in cystic fibrosis, chronic UTIs, and endocarditis
Physical and mechanical disruption [33]	Physical methods to break down biofilms and expose bacteria to treatments or the immune system	Ultrasound-mediated biofilm disruption, mechanical debridement, and laser therapy	Chronic wounds, surgical site infections, and implant-related infections
Surgical removal [34]	Complete or partial surgical removal of biofilm-infected tissues or devices to reduce microbial load and prevent recurrence	Debridement of infected tissues and removal of infected medical devices like prosthetics and catheters	Severe biofilm-associated infections such as infected joint prostheses, infected heart valves, and osteomyelitis
Biological therapies [35]	Use biological agents, such as bacteriophages, to target and eliminate biofilm-producing bacteria	Phage therapy, phage-derived enzymes (e.g., lysins), and enzyme-bacteriophage combination therapies	Treatment of multidrug-resistant biofilm infections in chronic wounds, diabetic foot ulcers, and burn infections
Nanoparticle-based drug delivery [36]	Utilization of nanoparticles to deliver antimicrobial agents directly to the biofilm site, enhancing drug penetration and reducing toxicity.	Silver nanoparticles, liposomal antibiotics, nanoparticle-based delivery of quorum-sensing inhibitors	Targeted treatment for biofilm infections in medical devices, chronic lung infections, and indwelling catheter infections
Preventive measures [37]	Development of materials and coatings that resist biofilm formation on medical devices and surfaces.	Biofilm-resistant coatings (e.g., silver-coated catheters, silicone implants), surface modification technologies	Prevention of biofilm formation on catheters, prosthetics, endotracheal tubes, and other medical devices

Future directions and research needs

Emerging trends in biofilm research emphasize the need for innovative strategies to overcome the challenges posed by biofilm-associated infections. One major area of focus is the advancement of genomic and proteomic technologies. These cutting-edge approaches allow researchers to gain deeper insights into the composition and behavior of biofilms, facilitating the identification of specific genes and proteins involved in biofilm formation and maintenance [38]. Systems biology, which integrates genomic data with computational modeling, enables the prediction of biofilm behavior under various environmental conditions. This holistic understanding is crucial for developing targeted interventions to disrupt biofilm formation and improve treatment outcomes [38]. A promising development is the identification of novel biomarkers for detecting biofilms. Healthcare providers can significantly improve diagnostic and therapeutic strategies by pinpointing molecular signatures that reliably indicate biofilms in clinical settings. Distinguishing between biofilm-associated infections and planktonic bacterial infections allows for more tailored treatments, leading to better patient outcomes. As research progresses, establishing biofilm-specific biomarkers will be essential for advancing the management of biofilm-related diseases [39]. Despite these advancements, several challenges remain. A major hurdle is the translation of laboratory findings into clinical practice. There is often a disconnect between academic research and industrial application, which can slow the development of practical solutions for biofilm-related healthcare issues. Bridging this gap requires collaboration between researchers, clinicians, and industry stakeholders to ensure that scientific discoveries apply to real-world scenarios [40]. Additionally, antibiotic resistance remains a critical challenge in biofilm management. The protective environment within biofilms allows microorganisms to evade traditional antimicrobial treatments, leading to persistent and hard-to-treat infections. Therefore, developing new therapeutic agents capable of penetrating and disrupting biofilms is essential to combat this growing issue [41,42]. Looking forward, several potential areas of research warrant further exploration. One is the development of new therapeutic agents specifically designed to target biofilms. Current research is focused on discovering compounds that either disrupt biofilm formation or enhance the susceptibility of biofilm-associated bacteria to existing antibiotics. Preclinical studies and well-designed clinical trials will be crucial in evaluating the safety and efficacy of these novel agents. Additionally, the emergence of personalized medicine in biofilm management offers promising possibilities. Tailoring therapies to individual patient profiles based on the specific biofilm characteristics and the host's immune response could revolutionize treatment strategies. Achieving this goal will require a multidisciplinary effort, integrating expertise from microbiology, immunology, and pharmacology to design customized treatment plans for patients suffering from biofilm-associated infections [5]. Future directions and research needs in biofilm management are summarized in Table 4.

**Table 4:** Future directions and research needs in biofilm management and therapy

Research area	Description	Key focus areas	Potential impact
Genomic and proteomic studies [43]	Investigating the genetic and proteomic profile of biofilm-forming organisms to identify new therapeutic targets	Whole-genome sequencing, biofilm-associated gene expression, and proteomic analysis	Development of targeted therapies, identification of biofilm-specific biomarkers, and better understanding of biofilm resilience
Novel biomarkers for biofilm detection [44]	Biomarkers indicating biofilm presence and activity are discovered for early and accurate diagnosis	Biofilm-specific proteins, metabolites, or genetic markers detectable in clinical samples	Improved diagnostic tools for timely detection and treatment of biofilm-associated infections
Challenges in antibiotic resistance [45]	Addressing the role of biofilms in developing antimicrobial resistance and finding new strategies to counteract it	Study of biofilm-mediated resistance mechanisms, and development of novel antibiotics or resistance-breaking compounds	Reduction in treatment failures due to antibiotic resistance, more effective use of existing antibiotics
Translational research [46]	Bridging the gap between laboratory research and clinical application to develop biofilm-targeted therapies that work in humans	Animal models of biofilm infections and clinical trials of biofilm-disrupting agents	Acceleration of new treatments into clinical use and improvement in patient outcomes
Innovative therapeutic agents [27]	Development of new drugs and biofilm-disrupting agents that target specific stages of biofilm formation	Antiquorum-sensing compounds, new antibiofilm drugs, and phage therapy advancements	Enhanced ability to disrupt biofilm formation, prevent chronic infections, and reduce antimicrobial resistance
Personalized medicine in biofilm management [47]	Tailoring treatment strategies based on individual patient factors and specific biofilm characteristics	Genomic profiling of infections and patient-specific treatment regimens	More effective, individualized treatments for biofilm-associated infections
Emerging technologies for biofilm control [48]	Exploration of novel technologies and materials for biofilm prevention and disruption on medical devices and surfaces	Nanotechnology, biofilm-resistant materials, and surface modification technologies	Prevention of biofilm formation in medical devices and reduction in hospital-acquired infections

## Conclusions

In conclusion, biofilms are a formidable challenge in managing chronic infections due to their complex structure and the protective barriers they create against host defenses and therapeutic interventions. The understanding of biofilm formation and its significant role in persistent infections underscores the need for advanced strategies to combat these resilient microbial communities. Despite current treatment limitations, emerging therapeutic approaches offer promising avenues for disrupting biofilms and enhancing infection management. Innovative strategies, including novel antimicrobial agents, physical disruption techniques, and biologically based therapies, hold the potential for improving clinical outcomes. Continued research and development are essential to overcoming the difficulties posed by biofilms, ultimately advancing the effectiveness of interventions and improving patient care in the face of chronic infections.
